# P-1691. Palliative medicine consultation does not reduce antimicrobial use during end-of-life care

**DOI:** 10.1093/ofid/ofae631.1857

**Published:** 2025-01-29

**Authors:** Hanna Y Akalu, Michael E DeWitt, Amlak Bantikassegn, Justin Brooten, Ryan C Maves

**Affiliations:** Wake Forest University School of Medicine, Winston-Salem, North Carolina; Atrium Wake Forest Baptist Health/ Wake Forest University School of Medicine, Winston-Salem, North Carolina; Wake Forest University School of Medicine, Winston-Salem, North Carolina; Wake Forest University School of Medicine, Winston-Salem, North Carolina; Wake Forest University School of Medicine, Winston-Salem, North Carolina

## Abstract

**Background:**

The role of antimicrobial drugs in end-of-life (EOL) care is uncertain, with the potential palliative benefit of antimicrobials in some settings being outweighed by potential limited benefit and added toxicity. In this study, we aimed to analyze the roles of palliative care consultation and code status change on antimicrobial prescribing practices at the EOL. We hypothesized that palliative medicine consultation (PMC) reduces antimicrobial use in patients receiving EOL care.

**Methods:**

This was a retrospective cohort study conducted on adult patients who were admitted to Wake Forest Baptist Health facilities between January-December 2019 and who were either transitioned to comfort care or discharged to hospice. Patients were stratified based on the presence or absence of PMC during their hospitalization. The primary outcome was the mean days of therapy (DOT) per patient before and after EOL care. DOTs were defined as a calendar day when a single systemic antimicrobial was administered. Antimicrobials given topically or prescribed solely for Pneumocystis or herpesvirus prophylaxis were excluded.

**Results:**

A total of 2242 patients were identified, of whom 621 received PM consultation during their hospitalization. Among those receiving PMC, there were a total of 3301 days of hospitalization and 4560 DOTs prior to the transition to EOL care, for a mean DOT/hospital day of 1.38 (95% CI 1.34-1.42). There were 6,563 hospital days and 11,466 DOTs among the non-PMC group, for a mean DOT/hospital day of 1.75 (1.72-1.78). Following transition to EOL care, antimicrobial use decreased rapidly, with 0.087 DOTs/hospital day in the PMC group (0.077-0.097) and 0.051 DOTs/hospital day (0.044-0.056) in the non-PMC group.

**Conclusion:**

In this observational study, antimicrobial drug use decreased markedly in patients transitioning to EOL care. However, the rate of antimicrobial use in EOL patients was not reduced by PMC and was greater than in those patients not receiving PMC, although this difference was not clinically substantial. Antimicrobial use was infrequent in EOL patients in our cohort. Future studies should evaluate the palliative benefit of antimicrobials in specific subgroups of patients receiving EOL care.

**Disclosures:**

**Ryan C. Maves, MD**, AiCuris: Grant/Research Support|Biotest: Grant/Research Support|GeoVax: Grant/Research Support|Shionogi: Advisor/Consultant|Shionogi: Honoraria|Sound Pharmaceuticals: Grant/Research Support

